# Presentation of the first international research network to foster high-quality clinical trials testing non-pharmacological interventions (TRACTION network)

**DOI:** 10.1136/bmjopen-2023-081864

**Published:** 2024-07-17

**Authors:** Ricardo J O Ferreira, Adriana Henriques, Rikke H Moe, Cristiano Matos, Anne-Therese Tveter, Nina Osteras, Paulo Nogueira, Andreia Silva Costa, Espen A Haavardsholm, Loreto Carmona, David Richards

**Affiliations:** 1 Nursing Research Innovation and Development Centre of Lisbon (CIDNUR), Nursing School of Lisbon (ESEL), Lisbon, Portugal; 2 Instituto de Saúde Ambiental (ISAMB), Faculdade de Medicina, Universidade de Lisboa, Lisboa, Portugal; 3 Rheumatology, Unidade Local de Saúde de Coimbra, Coimbra, Portugal; 4 QLV Research Consulting, Coimbra, Portugal; 5 Health Sciences Research Unit: Nursing (UICiSA:E), Nursing School of Coimbra (ESEnfC), Coimbra, Portugal; 6 Laboratório Associado TERRA, Faculdade de Medicina, Universidade de Lisboa, Lisboa, Portugal; 7 Instituto de Saúde Ambiental (ISAMB), Universidade de Lisboa Faculdade de Medicina, Lisboa, Portugal; 8 Center for Treatment of Rheumatic and Musculoskeletal Diseases (REMEDY), Diakonhjemmet Hospital, Oslo, Norway; 9 Department of Pharmacy, Instituto Politécnico de Coimbra Escola Superior de Tecnologia da Saúde de Coimbra, Coimbra, Portugal; 10 Centro de Investigação em Saúde Pública, Escola Nacional de Saúde Pública, Universidade NOVA de Lisboa, Lisboa, Portugal; 11 EPI Task-Force FMUL, Faculdade de Medicina, Universidade de Lisboa, Lisboa, Portugal; 12 Área Disciplinar Autónoma de Bioestatística (Laboratório de Biomatemática), Faculdade de Medicina, Universidade de Lisboa, Lisboa, Portugal; 13 Instituto de Salud Musculoesquelética (INMUSC), Madrid, Madrid, Spain; 14 Western Norway University of Applied Sciences, Bergen, Norway

**Keywords:** Clinical trials, Clinical Trial, THERAPEUTICS

## Abstract

Clinical trials are essential for evaluating the efficacy and safety of new treatments and health interventions. However, while pharmacological trials are well-established, non-pharmacological trials face unique challenges related to their complexity and difficulties such as recruitment, retention, intervention standardisation, selection of outcome measures and blinding of clinicians, participants and data collectors. This communication paper describes the objectives, implementation steps and bylaws of the ‘Trials foR heAlth Care inTerventIONs’ Network (TRACTION), established by an international multiprofessional task force of experts to foster high-quality non-pharmacological research, ultimately improving patient care and healthcare outcomes.

The TRACTION research network will provide information and resources through a collaborative hub for researchers, health professionals, patient research partners and stakeholders in diverse biomedical and healthcare areas, connecting people with different levels of expertise but with the same interests (eg, to evaluate the effect of non-pharmacological interventions, recruiting participants). This open network will support researchers in optimising trial design, participant recruitment, data management and analysis, and disseminating and implementing trial results.

The network will also facilitate specialisation training and provide educational materials and mentoring.

**STRENGTHS AND LIMITATIONS OF THIS STUDY**

- This viewpoint presents a novel international network for non-pharmacological clinical trials, aiming to foster interdisciplinary collaboration and provide comprehensive support for trial design, data management and dissemination, as well as training and knowledge sharing.
- The broad scope of the network encompasses a wide range of non-pharmacological interventions and healthcare settings, in different health disciplines and disease topics.
- Current activities and future expansion depend on securing additional funding, with the network infrastructure and full operational capabilities still under development. New contributors are invited to join and support this effort.

## Background

Clinical trials are necessary for developing new treatments and interventions, helping to clarify their efficacy and safety and improving the quality of care provided to patients.^1^ While pharmacological trials are well-established, there is a growing recognition of the need for improvements in non-pharmacological trials.^2^,^4^ Examples of non-pharmacological interventions are psychological therapies to improve depression,^5 6^ patient education to improve safety,^7^ or rehabilitation and physical activity promotion to enhance the quality of life and reduce disease activity.^8 9^

However, conducting non-pharmacological trials presents unique methodological and logistical challenges,^2 4^ for example, health provider, participant and research assessor blinding is often challenging or impossible to implement. An example of this complexity is in acupuncture trials, where ‘sham’ techniques, such as non-penetrating needling, shallow needling on points or non-points, or regular needling on non-points, are often employed. Yet, there is ongoing debate regarding acupuncture’s efficacy, distinguishing genuine therapeutic effects from the ‘placebo effect’ or potential influence of expectation bias.^10^ Such expectation biases can affect every interventional study but are often influenced by the nature of the intervention itself. Similarly, in trials involving cardiovascular medical devices, double-blind, randomised, controlled trials are not feasible and measures should be taken to minimise bias to the extent possible, such as selecting appropriate control group, the optimal length of follow-up or objective endpoints, among others.^11^ Non-pharmacological trials can face additional challenges compared with pharmaceutical trials regarding participant engagement and adherence^12^,^14^ and standardising the intervention’s delivery.^15^ These caveats can partially explain the poor description of methodological procedures in many of such trials.^16^ Often, these interventions are delivered by non-medical health professionals such as nurses, physiotherapists and psychologists, who might not have access to proper training opportunities to conduct such trials,^17^ also affecting their motivation and skills to engage in research.^18 19^ Additionally, these interventions generally face challenges in securing comparable financial and logistical support, especially when compared with pharmacological interventions backed by well-resourced entities.^20^

Altogether, these issues result in a systematically lower graded level of evidence supporting non-pharmacological interventions, which, on top of the complexity of these interventions, lead to limited implementation and availability to the general public. Approval agencies and organisations, still organised around delivering pharmaceutical trials, further uphold this cycle, requiring reflection and alternatives from the scientific community.^21 22^ The level of the problem is naturally dependent on the structures available and support for education and research in a given country.

To address some of these challenges, the European Economic Area (EEA) Grants funded the creation of a network called TRACTION (Trials foR heAlth Care inTerventIONs), under the Bilateral Relations Fund (FBR_OC2_33; https://www.eeagrants.gov.pt/en/) to support capacity building in Portugal (and in other countries) to perform non-pharmacological trials, leveraging on the expertise from International Scientists and tutored by a structure of excellence such as the REMEDY (Norway; https://en.remedy-senter.no/). This communication aims to describe the establishment of the TRACTION network, namely its objectives, framework, bylaws, educational materials and communication platform/website, to train and support international researchers from different professional backgrounds.

## The TRACTION network

This network proposes to bring together researchers, healthcare professionals, patient representatives and other stakeholders to collaborate and foster high-quality research in non-pharmacological healthcare interventions. TRACTION aims to:

- Support trial design and development, including the development of study protocols, selection of outcome measures and the identification of appropriate recruitment and retention strategies;
- Provide training and education resources for health professionals and researchers;
- Share experiences, novel ideas, strategies and tools for engaging and retaining participants in non-pharmacological trials, such as recruitment materials, communication tools and participant feedback mechanisms;
- Support data management and analysis with the development of data collection tools, data entry and statistical analysis;
- Foster dissemination and implementation of trial results, including developing publications, presentations and translating results into clinical practice.

The initial funding facilitated the establishment of the core concept, framework, bylaws and the development and piloting of a training workshop (free to participants), that is, the critical foundational steps. Future advancements will hinge on additional funding and the interest and collaborations fostered within the community, a process this communication aims to catalyse. Current information can be found here (https://www.traction.pt).

The TRACTION bylaws, available on the website, further detail the purpose, membership, advisory council, organisation, study activation, authorship and transition rules. The bylaws’ appendices include, among other things, guidelines to ensure ethical principles, define the eligibility criteria for participants, determine the study design, procedures for collecting and managing data, monitor the trial and report and disseminate the results.

The TRACTION network stands out due to its focus on non-pharmacological clinical trials. A key feature is the training for researchers in this specific area of trials. The first international workshop, lasting 1.5 days, took place in May 2023. This workshop comprised eight lectures, three keynote talks and three break-out group exercises to co-create and present a research project, all conducted in English (the official language). Eight sessions (box 1) were recorded and are freely available on ESEL’s YouTube channel, with subtitles in both English and Portuguese (https://www.youtube.com/playlist?list=PLRFn8o-LgXd3l4UOau80lrp24OBd4lerG). The workshop received 54 applications for the 20 available places, fully funded by the EEA Grant. Participants were primarily from Portugal, with five international attendees from Spain, Italy, the UK, Armenia and Tanzania. The participants had diverse professional backgrounds, including nursing, physiotherapy, occupational therapy, psychology and medicine.

Box 1 **Sessions of the TRACTION Workshop available on the YouTube**

1. Clinical trials and non-pharmacological studies: challenges and opportunities.

2. Defining FINER research questions for observational and experimental designs.

3. Deeper detail on interventional trial designs (parallel design, cross-over design, adaptative designs).

4. Sample selection, recruitment and randomisation techniques.

5. Introduction to complex interventions.

6. Ethics on a pragmatical perspective and involvement of patient research partners.

7. Understanding research data: intention-to-treat and related missing data and covariate adjustment.

8. Statistical hierarchy of endpoints and interim analyses.http://racslusofonia.org/

Importantly, TRACTION aims to extend its influence, particularly in Pan-American and Portuguese-speaking countries, through the ‘Lusophone Health Sciences Academic Network’ (http://racslusofonia.org/) and other bilateral protocols with international universities, such as the ones already established with ESEL (eg, Cape Verde or Macao).

### Conception

The steering group was formed by 11 researchers, predominantly from the ‘Nursing Research, Innovation and Development Centre of Lisbon’ (CIDNUR) at the ‘Nursing School of Lisbon’ (ESEL, Lisbon, Portugal), the ‘hosting institution’ and from the ‘Center for Treatment of Rheumatic and Musculoskeletal Diseases’ (REMEDY) at Diakonhjemmet Hospital (Oslo, Norway), the ‘mentoring institution’. These experts have nursing, physiotherapy and rheumatology as background. The group was completed by a fellow, with pharmacy and clinical trials data analysis as expertise (CM) and by two world-renowned experts: an epidemiologist and rheumatologist (LC) and a nurse and expert in ‘complex interventions in health’ (DR).

REMEDY is a Norwegian ‘Centre for Clinical Treatment Research’, established in 2022 with funding from the Research Council of Norway. Since 2008, the research environment has also been a Centre of Excellence recognised by the European Alliance of Associations for Rheumatology (EULAR). The centre aims to evolve patient care in the field of rheumatology and musculoskeletal diseases, by conducting clinical studies of pharmacological and non-pharmacological interventions that can potentially change clinical practice.

This group steered virtually four times a year. A selected team from CIDNUR visited the REMEDY centre to gain insights into its organisational structure and deliberate the fundamental components of the TRACTION network and its associated workshop. For TRACTION’s conception, established networks—though not specific to non-pharmacological trials—were consulted as foundational references, like the ‘Paediatric Rheumatology International Trials Organisation’ (PRINTO, https://www.printo.it/), the ‘Clinical Trials Network’ (https://thectnx.com/), the ‘European Clinical Research Infrastructure Network’ (ECRIN, https://ecrin.org/) or the ‘Portugal Clinical Trials’ (https://www.portugalclinicaltrials.com/en/).

### Envisaged features

The network will provide a collaborative and interdisciplinary environment where researchers, health professionals, patient research partners and other stakeholders can share resources, ideas and experiences and work together. The scope of the network is broad, encompassing a wide range of non-pharmacological interventions and healthcare settings, in different health disciplines and disease topics.

Among the main features envisaged for the platform/webpage that serves as a hub for the TRACTION network are a ‘Research collaboration search’ tool (to identify collaborators for projects), a ‘Q&A Forum’ (emphasising interdisciplinary collaboration and patient-centred research), a ‘Data Sharing Platform’, promoting open science principles and transparent reporting by providing a platform to share data (with additional requirement steps for certified users to have access to) and naturally, a ‘Knowledge Hub’, with videos (eg, lectures), best practices, guidelines, articles and other educational materials and tools to facilitate clinical trial.

### Regulatory compliance and support

The European Union Medical Device Regulation (EU MDR, 2017/745), effective from May 2021, replaces the previous Medical Devices Directive, aiming to enhance the safety, performance and quality of medical devices in the European market. This regulation introduces stringent standards and comprehensive requirements, demanding rigorous clinical evidence and extensive technical documentation. It also mandates continuous post-market surveillance and increased responsibilities for economic operators, ensuring accountability and traceability.

A pan-European network, like TRACTION, can significantly aid organisations in navigating these requirements while promoting knowledge sharing and dissemination of best practices among participants, enhancing the collective understanding of compliance strategies. This collaborative approach is particularly beneficial for small and medium-sized research groups, facing challenges in meeting the new regulatory demands.

## Future steps

The years ahead for TRACTION are earmarked by the ambition to lay a resilient foundation. Key endeavours include: (1) instituting a clear governance structure, prioritising effective management and participant engagement; (2) centralising patient voices by constituting a patient and public panel, ensuring their integral role from designing to disseminating trials; (3) charting a sustainability route that encompasses strategies for securing multifaceted funding and establishing liaisons with diverse stakeholders; (4) curating a repository of educational assets, focusing on areas pivotal to young researchers; (5) expanding the network’s outreach by fostering relationships with industry, healthcare entities and patient advocates; (6) actively inviting researchers to join the network, leveraging both modern digital means and traditional networking. Regarding this last task, at the moment, individuals or centres interested in joining or contributing to the TRACTION network can express their interest by sending an email to traction@esel.pt or by clicking the ‘Join Us’ button on our website. As soon as the network infrastructure is more advanced, candidates will need to complete a membership application form with information about their affiliation, type of stakeholder, area of expertise and interest, contact details, intended contributions, among other legal and organisational data. The TRACTION steering committee reviews all applications to ensure they align with the network’s goals and values, with notifications sent within a few weeks. Once approved, new members will have access to the network’s resources, according to the bylaws. TRACTION is designed to easily change and adapt based on what users need.

The resources provided for TRACTION are freely available with the intention of enabling the workshop to be conducted in as many locations as possible. A further edition in Portuguese is being planned.

### Patient and public involvement

Although the steering group of the TRACTION network did not include public or patient representatives in its initial development phase, it is planned to have active contributions from patient representative organisations in the following steps. We have designated mandatory places for patient representatives on the board. Future collaborations are anticipated with The European Patients’ Academy on Therapeutic Innovation (EUPATI) and local patient and public involvement groups already collaborating with CIDNUR and REMEDY centres.

### Limitations and challenges

While promising, the TRACTION network faces several challenges. Maintaining sustained engagement and participation from a diverse group of international researchers, health professionals and stakeholders is complex, especially when coordinating activities across different time zones and cultural contexts.

Another limitation is the reliance on continuous funding to support network activities, training workshops and resource development. Securing long-term financial support is crucial yet uncertain and competitive. Continuous funding applications, such as ‘The European Cooperation in Science and Technology (COST)’ actions, are being prepared to address this issue.

The network’s success depends on consistent methodologies across member institutions, but variability in resources and expertise can lead to inconsistencies.

Finally, adapting to evolving scientific, technological and regulatory landscapes is challenging, requiring continuous updates and compliance across diverse regulations.

## Closing remarks

The inception of the TRACTION network marks an important development in the realm of non-pharmacological trials in healthcare. Operating on pillars of collaboration, transparency and accountability, it extends an invitation to a broad spectrum of stakeholders, from researchers and health professionals to patient representatives, to collectively elevate the quality of patient care.

The TRACTION workshops or training activities can provide a unique opportunity for health professionals and researchers to develop their skills and knowledge in clinical trial design and implementation, through interactive talks and hands-on activities in order to gain insights into several aspects of non-pharmacological trials. These network activities will also facilitate networking and knowledge exchange among participants, contributing to a more comprehensive understanding of non-pharmacological research in all possible healthcare fields.

Overall, TRACTION seeks to advance the science of non-pharmacological interventions and ultimately improve patient care outcomes by providing more robust evidence and making these interventions available to the public and relevant stakeholders.
