# Supplementary material for: Presentation of the first international research network to foster high-quality clinical trials testing non-pharmacological interventions (TRACTION network)
Source: BMJ Open. 2024 Jul 17;14(7):e081864. doi: 10.1136/bmjopen-2023-081864 (PMC11256069; doi:10.1136/bmjopen-2023-081864)
Supplement: Supplementary file 1 [file bmjopen-2023-081864.reviewer_comments.pdf]

PEER REVIEW HISTORY

BMJ Open publishes all reviews undertaken for accepted manuscripts. Reviewers are asked to complete a checklist review form (**http://bmjopen.bmj.com/site/about/resources/checklist.pdf**) and are provided with free text boxes to elaborate on their assessment. These free text comments are reproduced below.

ARTICLE DETAILS

**TITLE (PROVISIONAL)**	Presentation of the first international research network to foster high-quality clinical trials testing non-pharmacological interventions (TRACTION network)
**AUTHORS**	Ferreira , Ricardo Jorge De Oliveira; Henriques, Maria Adriana; Moe, Rikke; Matos, Cristiano; TVETER, Anne Therese; Osteras, Nina; Nogueira, Paulo; Costa, Andreia; Haavardsholm, Espen A.; CARMONA, LORETO; Richards, David

VERSION 1 – REVIEW

**REVIEWER**	Descatha, Alexis<br>UNIV Angers Inserm , U1085 Irset, Ester Unit
**REVIEW RETURNED**	08-Jan-2024

**GENERAL COMMENTS**	It is an interesting network and should be known. However, I have some comments;<br>1/ I am a little confused by the aim of the paper. It is true that communication papers aim cover content that does not fit classic article types, but it should not present a report of a seminar that is the core of the paper as it appear with the program detailed. However, it is a possible topic that interest reader on two aspects: how to build such network, what goals and steps are needed. The second one is the core of the papers, but the first one is lacking. I feel, to interest readers, the project development of the network should be detailed: how founds were search (even we have founding, who were chosen and why, what timeline has been followed and choices. In extenso, what is between the background (chap 1) and Network (chap 2), and steps of project management of building such network.<br>2/ Non pharmacological trials in clinical settings are quite large and the authors should also clarify why they focus on nursing/ rheumatology and applicability outside8 of such themes?<br>3/ Instead of providing the program (what is the interest?), and evaluation of satisfaction (might be included as supplemental), a compete synthesis of the workshop is needed in the paper (at least global summary, each keynotes and example of project).<br>4/ Acknowledgments of participants are usually a good way not to only show a training course but a network . Authors contribution should include design of the network, funding...<br>5/ limitations of such network are also mandatory.

**REVIEWER**	Tanaka, Hideharu<br>Kokushikan University, emergency medicine
**REVIEW RETURNED**	25-Jan-2024

GENERAL COMMENTS	Thank you for the opportunity to review this paper<br>It encompassed a wide range of stakeholders, from extremely motivated researchers and health professionals to patient representatives, with the goal of building a network to improve the quality of patient care.<br>We recognized that the TRACTION workshop offers an extremely unique opportunity to support many of the non-pharmacological effects of treatments beyond the conventional pharmacological evaluation of health care professionals.<br>However, it also provided a unique opportunity to develop skills and knowledge in the design and conduct of clinical trials.<br>However, the anonymous digital satisfaction survey administered at the end of the workshop lacked objectivity due to the small number of participants, and it is considered to be in need of improvement.

REVIEWER	Cohen, Adam<br>Leiden University Medical Centre
REVIEW RETURNED	06-May-2024

GENERAL COMMENTS	The authors of this paper and the originators of the Traction network have addressed an important problem. The testing and approval of medicinal substances has reached a level of standardization and complexity that is unheard of and perhaps over the top. Devices or other medical interventions like surgery are still at a considerably different level. So this is commendable and worth reading about.<br>However, when reading the paper one gets the impression that the authors have padded up their excellent message to look like a research paper, which is unnecessary and in many ways distracting.<br>Therefore I suggest that a category for the paper is looked for that does not require this.<br>In this new version I have several suggestions<br>1. Refer to the MDR EU directive and the increased requirements this entails and how a pan EU network could help<br>2. Use more examples in the introduction. Acupuncture or homeopathy are less useful examples than psychotherapy or for instance renal denervation.<br>3. Explain how other organisations or individuals can join<br><br>So in summary, it is no sin to not have results at the moment but if the network was limited to an albeit successful course for 20 people I would say that it failed so the commentary should be ambitious and indicate roads towards that ambition. Then it is an important paper.

VERSION 1 – AUTHOR RESPONSE

Reviewer: 1
Prof. Alexis Descatha, UNIV Angers Inserm, CHU Angers Pôle A Vasculaire
Comments to the Author:
It is an interesting network and should be known. However, I have some comments;

COMMENT 1: I am a little confused by the aim of the paper. It is true that communication papers aim cover content that does not fit classic article types, but it should not present a report of a seminar that

is the core of the paper as it appear with the program detailed. However, it is a possible topic that interest reader on two aspects: how to build such network, what goals and steps are needed. The second one is the core of the papers, but the first one is lacking. I feel, to interest readers, the project development of the network should be detailed: how founds were search (even we have founding, who were chosen and why, what timeline has been followed and choices. In extenso, what is between the background (chap 1) and Network (chap 2), and steps of project management of building such network.

RESPONSE: Dear Prof. Alexis Descatha, we appreciate your comments that further improve our paper. We have now rewritten the manuscript and the "Chapter 3 – The first TRACTION workshop" report from the seminar was removed, as suggested also by other reviewers. Additionally, information on how to build the network (goals and steps) was briefly added.

ACTIONS TAKEN:

a) The last paragraph of the abstract was deleted:

"The network will also facilitate specialisation training and provide educational materials and mentoring. Here, we describe the structure, methods, content, and assessment of a pilot 1.5-day workshop in which 18 early-stage international researchers participated. All pedagogical materials used are shared, including eight video-recorded sessions, aiming to facilitate the replication of this workshop. This allows to accomplish the network goal that aims to facilitate specialization training.

b) The aim of the manuscript was changed (end of section 1, page 6):

This communication aims to describe the establishment of the TRACTION network, namely its objectives, framework, bylaws, educational materials and communication platform/website, to train and describe the structure, methods, content, and assessment of a pilot 1.5-day workshop and support international researchers.

c) The previous section "3. The first traction workshop" was completely deleted. As suggested by reviewers, we summarized the info into one paragraph, which was added into section 2 (page 7), reading as follows:

The TRACTION network stands out due to its focus on non-pharmacological clinical trials. A key feature is the training for researchers in this specific area of trials. The first international workshop with a 1.5-day duration took place in May 2023. This workshop was composed of eight lectures, three Keynote Talks, and three break-out group exercises, all run in English (the official language). Eight sessions were recorded and are freely available on the ESEL's YouTube, with subtitles in both English and Portuguese (https://www.youtube.com/playlist?list=PLRFn8o-LgXd3l4UOau80lrp24OBd4lerG). The workshop had 54 applications for the 20 places available, fully funded by the EEA Grant. Participants were mainly from Portugal (five were international: Spain, Italy, UK, Armenia, Tanzania) and with a nursing background (also a physiotherapist, an occupational therapist, a psychologist, and two physicians).

d) Edition of the second paragraph of the "Closing Remarks" (page 9):

"The TRACTION workshops or training activities can provided a unique opportunity for health professionals and researchers to develop their skills and knowledge in clinical trial design and implementation, through interactive talks and hands-on activities in order to gain insights into several aspects of non-pharmacological trials, which also facilitated networking and knowledge exchange among participants, contributing to a more comprehensive understanding of non-pharmacological research.

COMMENT 2: Non pharmacological trials in clinical settings are quite large and the authors should also clarify why they focus on nursing/ rheumatology and applicability outside of such themes?

RESPONSE: We understand that the focus on rheumatology and nursing might be perceived as predominant in the manuscript, as these are the key areas of expertise for a significant number of the founding members of this network: 4 out of 11 co-authors are nurses, and 6 out of 11 have rheumatology as their main area of activity. Additionally, the project was funded by a bilateral grant

with a Nursing research unit as the proponent and a Rheumatology research unit as the main partner. However, this network is not intended to be focused solely on these areas. To address this concern, we have clarified that the network is open to and useful for various multiprofessional activities and medical fields.
ACTIONS TAKEN: The following editions were done:

a) In the abstract:
The TRACTION research network will provide information and resources through a collaborative hub for researchers, health professionals, patient research partners, and stakeholders in diverse biomedical and healthcare areas, connecting people with different levels of expertise but with the same interests (…).

b) In the objective (page 7):
This communication aims to describe the establishment of the TRACTION network, namely its objectives, framework, bylaws, educational materials and communication platform/website, to train and support international researchers from different professional backgrounds.

c) In the first paragraph of section 2.2. (page 8):
The scope of the network is broad, encompassing a wide range of non-pharmacological interventions and healthcare settings, in different health disciplines and disease topics.

d) In the second paragraph of “Closing remarks” (page 9):
These network activities will also facilitate networking and knowledge exchange among participants, contributing to a more comprehensive understanding of non-pharmacological research in all possible healthcare fields.

COMMENT 3: Instead of providing the program (what is the interest?), and evaluation of satisfaction (might be included as supplemental), a compete synthesis of the workshop is needed in the paper (at least global summary, each keynotes and example of project).
RESPONSE: Dear Prof. Descatha, we appreciate your comments which further improve our paper. We have now rewritten the manuscript and the “Chapter 3 – The first TRACTION workshop” report from the seminar was removed, as suggested by two reviewers. In addition, the information on how to build the network and goals and steps were briefly added.
ACTIONS TAKEN:
a) We have rewritten the manuscript, and the section "Chapter 3 – The first TRACTION workshop" report from the seminar was removed.
b) The following text was added, including a “Box”:

The TRACTION network stands out due to its focus on non-pharmacological clinical trials. A key feature is the training for researchers in this specific area of trials. The first international workshop, lasting 1.5 days, took place in May 2023. This workshop comprised eight lectures, three Keynote Talks, and three break-out group exercises to co-create and present a research project, all conducted in English (the official language). Eight sessions (Box 1) were recorded and are freely available on ESEL’s YouTube channel, with subtitles in both English and Portuguese (https://www.youtube.com/playlist?list=PLRFn8o-LgXd3l4UOau80lrp24OBd4lerG). The workshop received 54 applications for the 20 available places, fully funded by the EEA Grant. Participants were primarily from Portugal, with five international attendees from Spain, Italy, the UK, Armenia, and Tanzania. The participants had diverse professional backgrounds, including nursing, physiotherapy, occupational therapy, psychology, and medicine.

Box 1 – Sessions of the TRACTION Workshop available on the YouTube
1 – Clinical trials and non-pharmacological studies: challenges and opportunities

2 – Defining FINER research questions for observational and experimental designs
3 – Deeper detail on Interventional trial designs (parallel design, cross-over design, adaptative designs)
4 – Sample selection, recruitment and randomization techniques
5 – Introduction to Complex interventions
6 – Ethics on a pragmatical perspective and Involvement of patient research partners
7 – Understanding Research Data: Intention-to-treat and related missing data and covariate adjustment
8 – Statistical hierarchy of endpoints and Interim analyses

COMMENT 4: Acknowledgments of participants are usually a good way not to only show a training course but a network.
RESPONSE: We agree that acknowledging participants effectively highlights the network's collaborative spirit. We have now included an acknowledgement section in the manuscript to recognize the contributions of all participants who attended the workshop.

ACTIONS TAKEN: The following paragraph was added in the "FOOTNOTES" section:
Acknowledgements
We would like to extend our gratitude to all the participants of the first TRACTION workshop held in May 2023. Your active engagement and valuable contributions were instrumental in the success of the workshop and in fostering a collaborative spirit within the network. We also thank the EEA Grant for fully funding the workshop, enabling us to bring together a diverse group of professionals from various countries and disciplines.

COMMENT 5: Authors contribution should include design of the network, funding...
RESPONSE: We have expanded the authors' contributions section to include details regarding the network's design and funding process.
ACTIONS TAKEN: That section was edited as follows:
Adriana Henriques, Ricardo J. O. Ferreira, and Andreia Costa conceptualised and designed the TRACTION network and secured funding. Rikke H. Moe, Anne-Therese Tveter, Nina Osteras, Paulo Nogueira, Cristiano Matos, Espen A. Haavardsholm, Loreto Carmona, and David Richards contributed to the design and development of the network.
Ricardo and Cristiano wrote the first draft of the manuscript, and all co-authors critically appraised and revised it, agreeing with its final version.

COMMENT 6: limitations of such network are also mandatory.
RESPONSE: Thank you for highlighting the importance of discussing the limitations of the TRACTION network. We have now included a section that addresses the various challenges and limitations associated with conducting non-pharmacological trials and the network itself.
ACTIONS TAKEN: Added a "Limitations" section in the manuscript:

a) Added a "Limitations" section in the manuscript, by the end of the "FUTURE STEPS" section:
Limitations and challenges
While promising, the TRACTION network faces several challenges. Maintaining sustained engagement and participation from a diverse group of international researchers, health professionals, and stakeholders is complex, especially when coordinating activities across different time zones and cultural contexts.
Another limitation is the reliance on continuous funding to support network activities, training workshops, and resource development. Securing long-term financial support is crucial yet uncertain

and competitive. Continuous funding applications, such as “The European Cooperation in Science and Technology (COST)” actions, are being prepared to address this issue.
The network's success depends on consistent methodologies across member institutions, but variability in resources and expertise can lead to inconsistencies.
Finally, adapting to evolving scientific, technological, and regulatory landscapes is challenging, requiring continuous updates and compliance across diverse regulations.

Reviewer: 2
Hideharu Tanaka, Kokushikan University
Comments to the Author:
Thank you for the opportunity to review this paper. It encompassed a wide range of stakeholders, from extremely motivated researchers and health professionals to patient representatives, with the goal of building a network to improve the quality of patient care. We recognized that the TRACTION workshop offers an extremely unique opportunity to support many of the non-pharmacological effects of treatments beyond the conventional pharmacological evaluation of health care professionals. However, it also provided a unique opportunity to develop skills and knowledge in the design and conduct of clinical trials.
COMMENT 1: (…) However, the anonymous digital satisfaction survey administered at the end of the workshop lacked objectivity due to the small number of participants, and it is considered to be in need of improvement.
RESPONSE: Dear Dr. Hideharu Tanaka, we appreciate your comments that further improve our paper. We acknowledge your concern regarding the objectivity of the satisfaction survey due to the small number of participants. In response to this and similar feedback from other reviewers, we have rewritten the manuscript. The report on the “Chapter 3 – The first TRACTION workshop” and the satisfaction survey results have been removed. Instead, we have focused on the broader aspects of the network's establishment, goals, and activities.

Reviewer: 3
Prof. Adam Cohen, Leiden University Medical Centre
Comments to the Author:
The authors of this paper and the originators of the Traction network have addressed an important problem. The testing and approval of medicinal substances has reached a level of standardization and complexity that is unheard of and perhaps over the top. Devices or other medical interventions like surgery are still at a considerably different level. So this is commendable and worth reading about.
COMMNET 1: However, when reading the paper one gets the impression that the authors have padded up their excellent message to look like a research paper, which is unnecessary and in many ways distracting.
Therefore I suggest that a category for the paper is looked for that does not require this.
In this new version I have several suggestions
RESPONSE: Dear Prof. Adam Cohen, thank you for your insightful comments and suggestions. We are very happy you found the manuscript of interest. As mentioned above, we have removed all the details about the workshop assessment, which were deviating the attention from the central message

COMMENT 2: Refer to the MDR EU directive and the increased requirements this entails and how a pan EU network could help.
RESPONSE: We agree that referring to the European Union Medical Device Regulation (EU MDR)

and its implications is important.
ACTIONS TAKEN: We have added a section addressing the EU MDR and the role of a pan-European network in meeting these new requirements, creating a new sub-section in the section “2. THE TRACTION NETWORK”:
2.3. Regulatory Compliance and Support
The European Union Medical Device Regulation (EU MDR, 2017/745), effective from May 2021, replaces the previous Medical Devices Directive, aiming to enhance the safety, performance, and quality of medical devices in the European market. This regulation introduces stringent standards and comprehensive requirements, demanding rigorous clinical evidence and extensive technical documentation. It also mandates continuous post-market surveillance and increased responsibilities for economic operators, ensuring accountability and traceability.
A pan-European network, like TRACTION, can significantly aid organisations in navigating these requirements while promoting knowledge sharing and dissemination of best practices among participants, enhancing the collective understanding of compliance strategies. This collaborative approach is particularly beneficial for small and medium-sized research groups, facing challenges in meeting the new regulatory demands.

COMMENT 3. Use more examples in the introduction. Acupuncture or homeopathy are less useful examples than psychotherapy or for instance renal denervation.
RESPONSE: We agree that including relevant examples will enhance the introduction's clarity and relevance. We have revised the introduction to briefly include an example of physical activity and exercise programs for managing cardiovascular diseases to better illustrate the scope and importance of non-pharmacological interventions. Please note that in the first paragraph of the introduction we already mention several examples of non-pharmacological interventions: “Examples of non-pharmacological interventions are psychological therapies to improve depression [5,6], patient education to improve safety [7], or rehabilitation and physical activity promotion to enhance the quality of life and reduce disease activity [8,9].”
ACTIONS TAKEN: The following paragraph was edited in the introduction:
“Yet, there is ongoing debate regarding acupuncture’s efficacy, distinguishing genuine therapeutic effects from the "placebo effect" or potential influence of expectation bias [10]. Such expectation biases can affect every interventional study but are often influenced by the nature of the intervention itself. Similarly, in trials involving cardiovascular medical devices, double-blind, randomised, controlled trials are not feasible and measures should be taken to minimize bias to the extent possible, such as selecting appropriate control group, optimal length of follow-up, or objective endpoints, among others [REF].”

REF: Zannad F, Stough WG, Piña IL, Mehran R, Abraham WT, Anker SD, De Ferrari GM, Farb A, Geller NL, Kieval RS, Linde C, Redberg RF, Stein K, Vincent A, Woehrle H, Pocock SJ. Current challenges for clinical trials of cardiovascular medical devices. Int J Cardiol. 2014 Jul 15;175(1):30-7. doi: 10.1016/j.ijcard.2014.05.021. Epub 2014 May 16. PMID: 24861254.

COMMENT 4. Explain how other organisations or individuals can join
RESPONSE: Thank you for your valuable suggestion. We agree that providing clear information on how other organizations or individuals can join the TRACTION network will enhance the manuscript's utility and relevance. We should note that we are not yet fully promoting applications as we need further funding to develop a proper platform and fund board meetings to prepare all details adequately.
ACTIONS TAKEN: The following sentence was added in the “3 FUTURE STEPS” section:
The years ahead for TRACTION are earmarked by the ambition to lay a resilient foundation. Key endeavours include: i) Instituting a clear governance structure, prioritising effective management and participant engagement; ii) Centralizing patient voices by constituting a patient and public panel, ensuring their integral role from designing to disseminating trials; iii) Charting a sustainability route

that encompasses strategies for securing multifaceted funding and establishing liaisons with diverse stakeholders; iv) Curating a repository of educational assets, focusing on areas pivotal to young researchers; v) Expanding the network's outreach by fostering relationships with industry, healthcare entities, and patient advocates; vi) Actively inviting researchers to join the network, leveraging both modern digital means and traditional networking. Regarding this last task, at the moment, individuals or centres interested in joining or contributing to the TRACTION network can express their interest by sending an email to traction@esel.pt or by clicking the "Join Us" button on our website. As soon as the network infrastructure is more advanced, candidates will need to complete a membership application form with information about their affiliation, type of stakeholder, area of expertise and interest, contact details, intended contribution, among other legal and organisational data. The TRACTION steering committee reviews all applications to ensure they align with the network's goals and values, with notifications sent within a few weeks. Once approved, new members will have access to the network's resources, according to the bylaws.

COMMENT 5: So in summary, it is no sin to not have results at the moment but if the network was limited to an albeit successful course for 20 people I would say that it failed so the commentary should be ambitious and indicate roads towards that ambition. Then it is an important paper.
RESPONSE: Thank you for your insightful comment, which aligns with the suggestion of previous reviewers. To ensure the paper aligns with the type of article and interest of the audience, we have removed the detailed section on the workshop assessment. We believe this refocuses the manuscript on the broader goals and future steps and ambition of the TRACTION network.

VERSION 2 – REVIEW

**REVIEWER**	Descatha, Alexis<br>UNIV Angers Inserm , U1085 Irset, Ester Unit
**REVIEW RETURNED**	26-Jun-2024

**GENERAL COMMENTS**	The authors improved the manuscript.

**REVIEWER**	Cohen, Adam<br>Leiden University Medical Centre
**REVIEW RETURNED**	12-Jun-2024

**GENERAL COMMENTS**	Thanks for dealing wih the comments of the reviewers
